# Intrauterine L-NAME Exposure Weakens the Development of Sympathetic Innervation and Induces the Remodeling of Arterial Vessels in Two-Week-Old Rats

**DOI:** 10.3390/ijms222212327

**Published:** 2021-11-15

**Authors:** Ekaterina K. Selivanova, Anastasia A. Shvetsova, Anna A. Borzykh, Dina K. Gaynullina, Oxana O. Kiryukhina, Elena V. Lukoshkova, Viktoria M. Potekhina, Vladislav S. Kuzmin, Olga S. Tarasova

**Affiliations:** 1 Department of Human and Animal Physiology, Faculty of Biology, M.V. Lomonosov Moscow State University, 119234 Moscow, Russia; selivanova@mail.bio.msu.ru (E.K.S.); anastasiashvetsova92@gmail.com (A.A.S.); dina.gaynullina@gmail.com (D.K.G.); vm-potekhina@yandex.ru (V.M.P.); ku290381@mail.ru (V.S.K.); 2 Laboratory of Exercise Physiology, State Research Center of the Russian Federation, Institute for Biomedical Problems, Russian Academy of Sciences, 123007 Moscow, Russia; borzykh.anna@gmail.com; 3 Laboratory for the Study of Information Processes at the Cellular and Molecular Levels, Institute for Information Transmission Problems, Russian Academy of Sciences, 119333 Moscow, Russia; kcyu@yandex.ru; 4 Laboratory of Experimental Pathology of the Heart, National Medical Research Center of Cardiology, Ministry of Health of the Russian Federation, 121552 Moscow, Russia; elena.lukoshkova@gmail.com

**Keywords:** nitric oxide, sympathetic innervation, early postnatal development, vasculature, blood pressure

## Abstract

Nitric oxide (NO) has been shown to stimulate differentiation and increase the survival of ganglionic sympathetic neurons. The proportion of neuronal NOS-immunoreactive sympathetic preganglionic neurons is particularly high in newborn rats and decreases with maturation. However, the role of NO in the development of vascular sympathetic innervation has never been studied before. We tested the hypothesis that intrauterine NO deficiency weakened the development of vascular sympathetic innervation and thereby changed the contractility of peripheral arteries and blood pressure level in two-week-old offspring. Pregnant rats consumed NOS inhibitor L-NAME (250 mg/L in drinking water) from gestational day 10 until delivery. Pups in the L-NAME group had a reduced body weight and blood level of NO metabolites at 1–2 postnatal days. Saphenous arteries from two-week-old L-NAME offspring demonstrated a lower density of sympathetic innervation, a smaller inner diameter, reduced maximal active force and decreased α-actin/β-actin mRNA expression ratio compared to the controls. Importantly, pups in the L-NAME group exhibited decreased blood pressure levels before, but not after, ganglionic blockade with chlorisondamine. In conclusion, intrauterine L-NAME exposure is followed by the impaired development of the sympathetic nervous system in early postnatal life, which is accompanied by the structural and functional remodeling of arterial blood vessels.

## 1. Introduction

Nitric oxide (NO) is an important regulatory molecule in mammalian organisms. Physiologically relevant NO is generated by two constitutive nitric oxide synthase isoforms: neuronal (nNOS) and endothelial (eNOS) [1,2]. nNOS is predominantly expressed in the central and peripheral nervous system, whereas eNOS is the most typical of the vascular endothelium. In a healthy organism, NO plays a key role in reducing vascular tone and blood pressure level and serves as a neurotransmitter and retrograde messenger as well as modulating synaptic plasticity [1,2,3].

In early postnatal ontogenesis, the regulation of the circulatory system has many unique characteristics compared to adult organisms, one of which is the augmented role of NO in vasomotor control [2,4]. Such a high level of NO production/action is important for maintaining low blood pressure in the developing cardiovascular system [5,6] and preventing the overstretching of and damage to the immature heart and vasculature. A gradual rise in blood pressure as the body develops is associated with a decrease in NO vasodilator effects in most vascular beds [2,5]. Another mechanism that contributes substantially to the postnatal pressure rise is the development of sympathetic vasomotor innervation [7,8]. Sympathetic innervation of the rat vasculature only develops after birth and gradually becomes mature within the first postnatal month [9,10]. In addition to the acute vasoconstrictor effect, sympathetic nerves exert trophic action by promoting the growth of vascular smooth muscle cells, their differentiation into contractile phenotype, and consequently, vessel remodeling [11,12,13]. Of note, chronic sympathectomy reduces the maximum contractility of peripheral arteries in rats [14].

Data in the literature suggest the role of NO in the control of sympathetic innervation development. Cell culture studies have demonstrated that NO can stimulate differentiation/neurite growth and increase the survival of nerve cell precursors and sympathetic neurons [15,16,17,18]. The expression of nNOS in sympathetic preganglionic neurons is much higher in newborn rats and decreases with age [19]. Therefore, excessive perinatal NO production may be beneficial for the development of vascular sympathetic innervation in early postnatal ontogenesis. In contrast, NO deficiency delays sympathetic innervation development. Importantly, a decrease in NO bioavailability in the developing organism may be the result of maternal preeclampsia [20,21], which affects up to 5% of all pregnancies and has adverse outcomes on the offspring circulatory system [22,23]. In particular, neonatal arterial hypotension is common in infants born from preeclamptic mothers [24], although it is not yet clear whether this is due to the underdevelopment of sympathetic vascular control.

In the present study, we tested the hypothesis that NO deficiency in the developing organism weakens the formation of vascular sympathetic innervation, which may result in the decreased contractility of peripheral arteries and the lowering of blood pressure levels. Intrauterine NO deficiency was achieved through the chronic supplementation of N^ω^-nitro-L-arginine methyl ester (L-NAME) to pregnant females [25,26,27]. L-NAME has equally high potency towards eNOS and nNOS [28]. Experiments were carried out on two-week-old offspring because vascular sympathetic innervation has already appeared but remains immature and continues to develop at this age [9,10,29].

## 2. Results

### 2.1. Characteristics of L-NAME Treatment Model

#### 2.1.1. Pregnant Females

**Figure 1:**
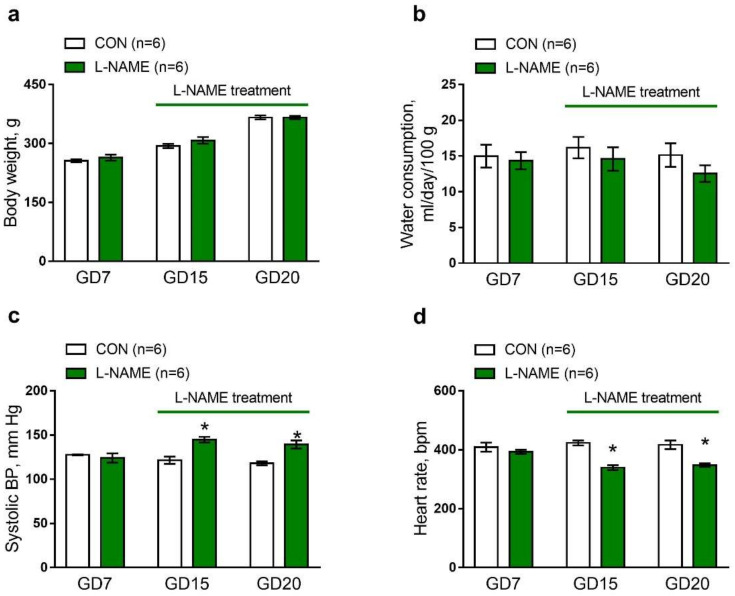
Characteristics of pregnant female rats in the control (CON) and L-NAME groups. (**a**) Body weight gain during the pregnancy. (**b**) Water consumption by dams in the control and L-NAME groups. (**c**–**d**) Systolic blood pressure (BP, **c**) and heart rate (**d**) during the pregnancy, before (GD7), and during L-NAME treatment (GD15 and GD20); GD, gestation day; *n*, number of females. Data are presented as the mean ± S.E.M. * *p* < 0.05 compared to the control on the same day of pregnancy (unpaired Student’s *t*-test).

The body weights of females in the control group and L-NAME group were almost similar at the seventh gestation day (GD7, Figure 1a). Consumption of L-NAME solution (250 mg/L) from GD10 until delivery did not affect the weight gain of females (Figure 1a). Similarly, relative water consumption by pregnant females was not changed by L-NAME treatment (Figure 1b). The average daily dose of L-NAME consumed by females of the experimental group from GD10 to delivery was 37 mg/kg.

Systolic blood pressure and heart rate were not different between the control and L-NAME groups before L-NAME treatment (Figure 1c,d, GD7). During L-NAME consumption, dams presented an increase in systolic blood pressure (Figure 1c, GD15 and GD20). As compensation for high blood pressure, heart rate values of L-NAME-treated dams decreased in comparison to the control group at GD15 and GD20 (Figure 1d).

Notably, the consumption of L-NAME led to a decrease in the content of NO metabolites in the blood serum of females on GD18-19. Any notable changes in several metabolic parameters, such as triglycerides and total cholesterol, were not observed after L-NAME consumption (Table 1).

#### 2.1.2. Offspring

**Figure 2:**
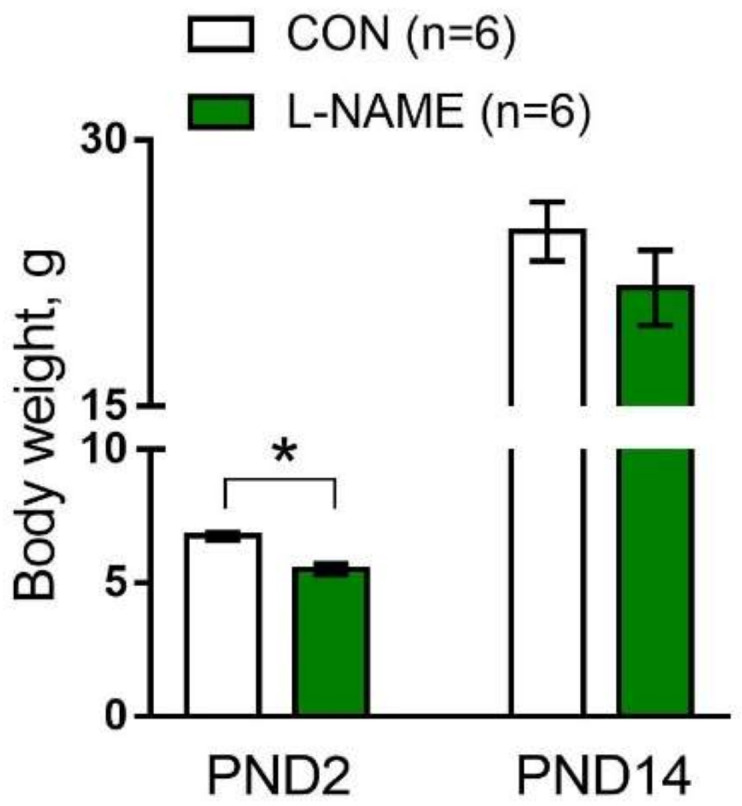
Body weights of the offspring of control (CON) and L-NAME-treated females at the 2nd (PND2) and the 14th postnatal days (PND14). Data are presented as the mean ± S.E.M., *n*, number of litters. * *p* < 0.05 (unpaired Student’s *t*-test).

The duration of pregnancy was 21–22 days in both groups of females. The number of pups per litter was not different between L-NAME and control females (12 ± 1 and 11 ± 1, respectively, *p* > 0.05). Offspring born from L-NAME-treated females (“L-NAME offspring” in the following description) exhibited a reduced body weight at postnatal day 2 (PND2) but reached control values by PND14 (Figure 2). The content of NO metabolites in blood serum was strongly reduced in L-NAME offspring on PND2 and recovered to the level observed in the control group by PND14 (Table 2). The contents of triglycerides and total cholesterol were not different between two groups of pups on either PND2 or PND14 (Table 2).

Further studies of isolated arteries and blood pressure recording were performed using 14–16-day-old male pups (at least 14 days after the termination of L-NAME treatment). They are referred to as two-week-old pups in the following text.

### 2.2. Studies of Two-Week-Old Offspring Arteries

#### 2.2.1. The Density of Sympathetic Innervation in Saphenous Artery Wall

To identify whether NO deficiency affects the development of sympathetic innervation, we evaluated the density of adrenergic fiber plexus in isolated saphenous arteries in two-week-old pups. This resistance-type small artery predominantly provides blood flow to the skin of the foot and presents dense sympathetic innervation in adult rats [9,10].

**Figure 3:**
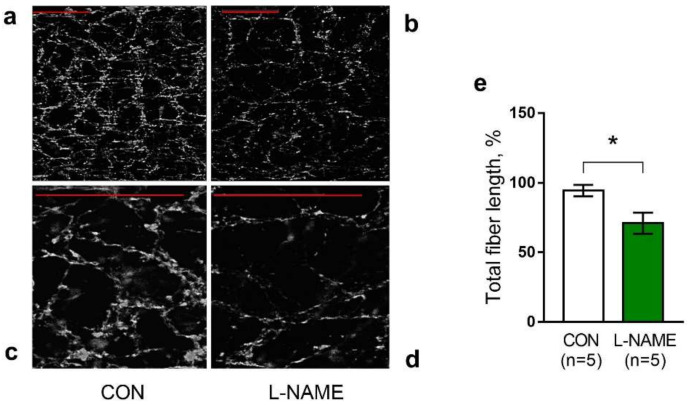
The density of sympathetic innervation of the saphenous artery is reduced in two-week-old L-NAME offspring. (**a**–**d**) Representative images of the glyoxylic-acid-stained plexus of adrenergic nerve fibers in the saphenous artery walls of control (**a**,**c**) and L-NAME (**b**,**d**) offspring (scale bar: 50 μm). (**e**) The total length of adrenergic fibers in the saphenous artery of the offspring from the control and L-NAME groups. *n*, number of animals. Data are presented as the mean ± S.E.M., * *p* < 0.05 (unpaired Student’s *t*-test).

Sympathetic innervation density was estimated by the total length of the skeletonized nerve fibers [30]. Figure 3 demonstrates that the total adrenergic nerve fiber length was significantly reduced in arteries from L-NAME offspring (Figure 3a,c,e) in comparison to the controls (Figure 3b,d,e).

#### 2.2.2. Functional Characteristics of Saphenous Artery

As the next step of our study, the functional characteristics of two-week-old offspring saphenous artery were examined. The inner diameters of relaxed saphenous arteries (0.9d_100_, see Section 4 for details) were reduced in L-NAME rats compared to controls: 276 ± 6 μm (*n* = 17) vs. 296 ± 8 μm (*n* = 18) (*p* < 0.05, unpaired Student’s *t*-test).

Contractile responses of saphenous arteries were induced by methoxamine, an agonist of α_1_-adrenoceptors, which are the main target of noradrenaline released from sympathetic nerves [31]. Importantly, methoxamine is not taken up by sympathetic nerve terminals [32], which allowed us to compare α_1_-adrenergic contractile responses of arteries with different adrenergic fiber densities.

**Figure 4:**
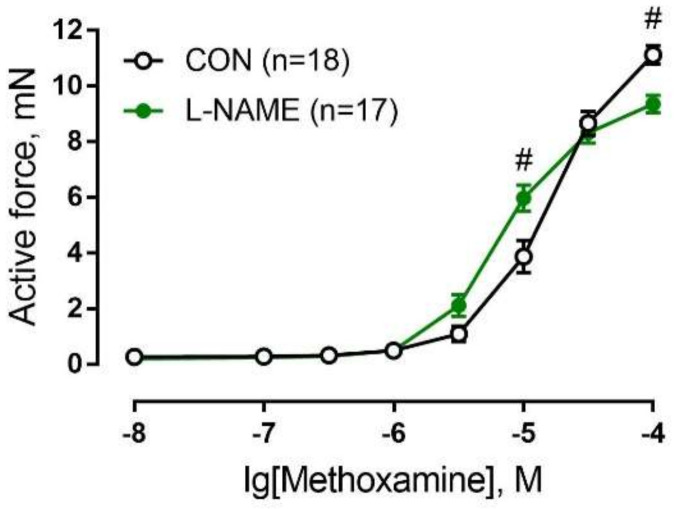
Concentration-dependent contractile response of saphenous arteries of two-week-old offspring from the control (CON) and L-NAME groups to the α_1_-adrenoceptor agonist methoxamine. Data are presented as the mean ± S.E.M., *n*, number of animals. # *p* < 0.05 compared to respective value in the control group (two-way ANOVA followed by Šidák’s post hoc analysis).

The comparison of concentration–response relationships revealed non-uniform changes in arterial contractile responses to different concentrations of methoxamine (Figure 4). The responses to moderate concentrations of methoxamine were higher in the L-NAME group, whereas in contrast, the response to the highest concentration was lower compared to the control.

Such alterations of contractile responses suggest increased arterial sensitivity but decreased maximum contractility to methoxamine in the L-NAME group. To quantify these parameters, we fitted concentration–response relationships to a sigmoidal dose–response equation. The sensitivity of saphenous arteries to the α_1_-adrenoceptor agonist methoxamine was increased in L-NAME offspring (Table 3). Additionally, the maximum active force developed by the saphenous artery of the L-NAME group was reduced compared to the control (Table 3).

#### 2.2.3. Expressed mRNA Levels

**Figure 5:**
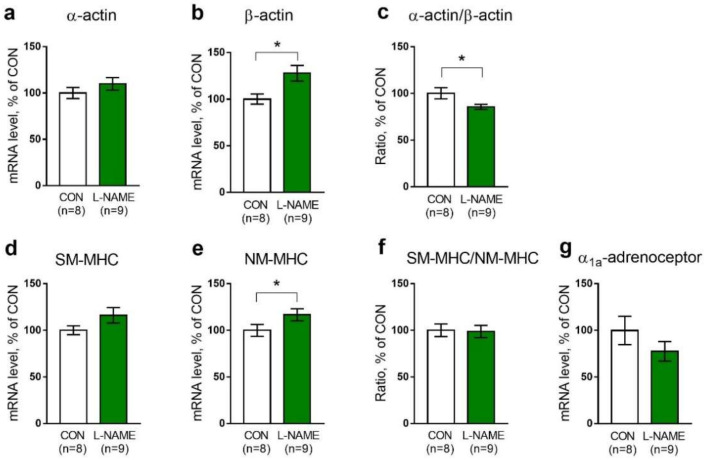
Relative mRNA levels of α-actin (**a**), β-actin (**b**), α-actin/β-actin ratio (**c**), smooth muscle myosin heavy chain isoform (SM-MHC) (**d**), non-muscle myosin heavy chain isoform (NM-MHC) (**e**), SM-MHC/NM-MHC ratio (**f**) and α_1a_-adrenoceptor (**g**) in the saphenous artery of control (CON) or L-NAME offspring. Data are presented as the mean ± S.E.M., *n*, number of animals. * *p* < 0.05 (unpaired Student’s *t*-test). Data are normalized to the mRNA content of RPLP0 in the same tissue sample; the mean value in the control group was taken as 100%.

Furthermore, we evaluated the expression levels of genes that might characterize the maturity of arterial smooth muscle (see the Section 4, Table 4). The relative mRNA expression levels of smooth muscle α-actin were not different between arterial samples from control and L-NAME offspring, whereas the expression levels of non-muscle β-actin were increased in arteries from L-NAME pups (Figure 5a,b). As a result, saphenous arteries from L-NAME pups exhibited a decrease in the α-actin/β-actin ratio in comparison to the control (Figure 5c). Smooth muscle myosin heavy chain isoform (SM-MHC) mRNA levels were not different between the pups from the control and L-NAME groups (Figure 5d). The expression of non-smooth muscle heavy chain isoform (NM-MHC) was slightly increased in the arteries from L-NAME offspring (Figure 5e). The SM-MHC/NM-MHC ratio was not different between the two experimental groups (Figure 5f). The mRNA levels of α_1a_-adrenoceptor, a subtype that is preferentially activated by methoxamine [33], were not different in arterial samples of L-NAME and control pups (Figure 5g).

### 2.3. Systemic Cardiovascular Parameters of Two-Week-Old Offspring

In order to clarify whether the reduced density of sympathetic innervation of peripheral arteries was associated with the altered control of systemic hemodynamics, mean arterial pressure and heart rate were measured in anesthetized two-week-old pups.

**Figure 6:**
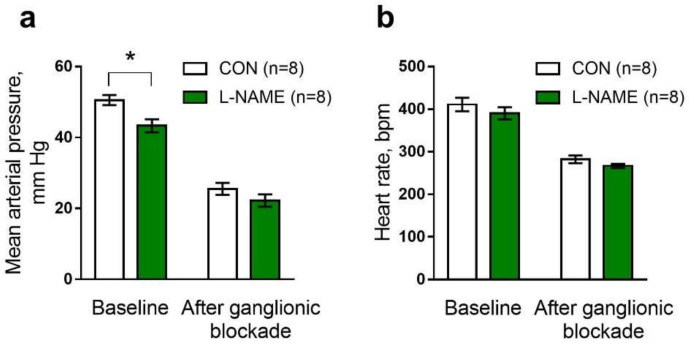
Systemic cardiovascular parameters of anesthetized two-week-old offspring before and after the ganglionic blockade. Mean arterial pressure (**a**) and heart rate (**b**) values before (baseline) and after ganglionic blockade (chlorisondamine, 2.5 mg/kg) in pups from control (CON) and L-NAME groups. Data are presented as the mean ± S.E.M., *n*, number of animals. * *p* < 0.05 (unpaired Student’s *t*-test).

The L-NAME offspring had lower baseline levels of mean arterial pressure (Figure 6a) in comparison to the control group. Basal heart rate values of the L-NAME and control offspring were similar (Figure 6b).

To evaluate the contribution of the sympathetic nervous system to the regulation of neurogenic vascular tone, we performed a ganglionic blockade with chlorisondamine (2.5 mg/kg, i.v.). Chlorisondamine caused a drop in mean arterial pressure in both groups, and the mean arterial pressure was no longer different between groups (Figure 6a). Notably, ganglionic blockade led to a similar decrease in heart rate in pups from control and L-NAME groups (Figure 6b).

## 3. Discussion

### 3.1. L-NAME Treatment Leads to NO Deficiency in Dams and Their Offspring

We showed that the body weight gain in dams was not affected by the administration of L-NAME from GD10 until a delivery. Notably, L-NAME treatment was able to cause a rise in systolic blood pressure in dams, even though the dose used in this experiment was relatively lower than that reported in the literature [25,26,34,35,36,37]. A number of studies have shown that a similar effect of L-NAME is observed at higher doses [25,26,27]. Consistent with previous research [38], blood pressure elevation was observed in L-NAME-treated females despite a compensatory decrease in heart rate, which suggests an increase in peripheral vascular resistance. Importantly, peripheral vasoconstriction upon L-NAME treatment may be due to the inhibition of not only vascular eNOS [39], but also the nNOS of brain stem neurons responsible for cardiovascular control [3]. Assuming a uniform distribution of L-NAME in tissues, it can be expected that it will inhibit eNOS and nNOS in the female body to the same extent. The same could take place in fetuses during intrauterine L-NAME exposure and in pups during a few days after birth.

Vasoconstriction and limited uteroplacental perfusion may lead to intrauterine growth restriction of the fetus [23,40]. Our study demonstrated that L-NAME offspring had a reduced birth body weight but reached the control values by the 14th day of postnatal life. Similarly, several studies have shown a decrease in newborn rat body weight and its recovery by the second week of postnatal life [37,41]. Such a rapid recovery in body weight is probably due to sufficient nutrient uptake after birth. Notably, L-NAME administration may induce liver injury and, consequently, lipid metabolism disturbance [42]. However, this was not observed in our work: blood contents of triglycerides and total cholesterol were not different between control and L-NAME-treated females, as well as between respective groups of offspring.

The relevance of our NO deficiency model was also confirmed by a decrease in the blood content of nitric oxide metabolites in L-NAME-treated females. The concentration of nitric oxide metabolites in blood sera of the offspring born from L-NAME-treated rats was also significantly reduced compared to controls soon after birth (PND2), which can be explained by the ability of L-NAME to cross the placenta, in addition to maternal NO deficiency [43]. Previously, we have shown that the blood contents of nitric oxide metabolites in newborn rats were reduced by two-fold higher doses of L-NAME [27]. At the age of two weeks (PND14), the blood content of NO metabolites in L-NAME offspring returned to control levels. However, due to the great reduction in NO metabolite contents in L-NAME offspring at birth (one-quarter of the control), we assume that NO deficiency in their tissues persisted for some time. Therefore, NO production was suppressed in L-NAME offspring during the second half of prenatal development, and, presumably, several days after birth. Therefore, the used experimental model was relevant for the proposed goal of the study.

### 3.2. Intrauterine L-NAME Exposure Is Associated with a Reduced Density of Sympathetic Innervation in Peripheral Arteries

In addition to its important vasomotor role, NO was shown to promote differentiation and increase the survival of nerve cell precursors and sympathetic neurons [15,16,17,18,44]. A recent study showed that the number of nNOS-immunoreactive sympathetic preganglionic neurons in rats gradually decreased within the first month of life [19], pointing to the role of NO in the regulation of sympathetic innervation development in early postnatal ontogenesis.

To assess whether NO deficiency would influence the development of sympathetic innervation, the density of sympathetic nerve fibers was evaluated in the isolated saphenous artery. Notably, in adult rats, the saphenous artery exhibits dense sympathetic innervation and develops strong contractile responses to the stimulation of sympathetic nerves [10,45]. According to our previously published data, developmental alterations of several regulatory mechanisms (the activity of Rho-kinase and the NO pathway) observed in the saphenous artery are associated with corresponding changes at the level of blood pressure control [5,8]. Therefore, we consider the saphenous artery to be a representative artery of the peripheral circulation.

Our data demonstrate that the total length of adrenergic fibers in the saphenous artery of two-week-old pups born from L-NAME-treated females is reduced compared to controls. Impaired vascular innervation development can be a long-lasting effect of L-NAME exposure, which is preserved after the disappearance of the substance from pup’s tissues. Thus, NO may control the development of vascular sympathetic innervation during early postnatal ontogenesis. Notably, the sympathetic nerve plexus in the peripheral arteries of two-week-old rats is still developing [9,10,29]. Therefore, sympathetic innervation injuries and the associated remodeling of blood vessels could be more easily detected at this age compared to ages of 1 month and older, at which time the periarterial sympathetic nerve plexus has already been formed.

### 3.3. Intrauterine L-NAME Exposure Is Associated with the Delayed Maturation of Peripheral Arteries

It is well known that the trophic action of sympathetic nerves promotes the growth and differentiation of vascular smooth muscle cells and vessel remodeling [12,46]. Our data suggest that trophic nerve influences were reduced in the sparsely innervated arteries of L-NAME offspring. Next, we will consider the reasons for such a point of view.

We have shown that saphenous arteries from two-week-old L-NAME offspring have a smaller inner diameter and maximum contractility, the latter often correlating with reduced media thickness [47]. The assumption that such changes are due to a weakened trophic sympathetic influence is supported by the results of previous studies, which showed similar changes in arterial characteristics after chronic sympathectomy [14,46].

Trophic action of the sympathetic nerves was shown to increase the expression of smooth-muscle-specific α-actin and myosin [12,13], promoting the differentiation of vascular smooth muscle cells into contractile phenotypes. However, despite our expectations, we did not observe a decrease in mRNA expression of these genes in the arteries of L-NAME offspring. Presumably, although the nerve density was reduced, trophic nerve influence was sufficient to maintain the expression of α-actin and myosin genes. Previously, we demonstrated decreased mRNA contents of α-actin and smooth-muscle myosin heavy chain isoforms in the aorta of L-NAME offspring compared to control pups [27]. Such a discrepancy between our present and previous data may be due to the lower dose of L-NAME (37 mg/kg vs. 78 mg/kg), the older age of the studied rat pups (2 weeks vs. PND1), and the usage of different objects (saphenous artery vs. aorta). Additionally, in the present study, we observed a moderate decrease in the ratio of smooth muscle α-actin and structural β-actin mRNA expression. In addition to the reduction in media thickness suggested above, this might be another reason for the decrease in maximal active force developed by the saphenous artery in L-NAME rat pups.

The arterial sensitivity to α_1_-adrenoceptor agonist methoxamine in L-NAME offspring was considerably higher than that in the control group. We considered that such arterial hypersensitivity was due to the altered response of smooth muscle, not by the attenuated anticontractile effect of the endothelium. In a one- or two-week-old rat saphenous artery, the endothelium weakens the effects of vasoconstrictors due to the release of NO [4]. In our study, NOS inhibitor L-NNA similarly increased the contractile responses of saphenous arteries in control and L-NAME rats (Figure S1). Importantly, in the presence of L-NNA, arterial sensitivity to methoxamine was still elevated in L-NAME offspring compared to controls: pD2 values were 5.83 ± 0.08 and 5.28 ± 0.08, respectively (*p* < 0.05, unpaired Student’s *t*-test). This points to the higher sensitivity of smooth muscle cells to methoxamine in L-NAME offspring compared to controls.

The arteries of L-NAME offspring are more sensitive to methoxamine because of their sparse sympathetic innervation. Indeed, the augmented sensitivity of chronically denervated arteries to adrenoceptor agonists was shown in several studies [14,48,49,50]. Commonly, post-denervation hypersensitivity occurs without changes in total density or the affinity of post-junctional α_1A_-adrenoceptors [51,52], which have the highest affinity to methoxamine compared to B and D subtypes [33]. Presumably, the same took place in two-week-old offspring of L-NAME-treated females, because the content of α_1A_-adrenoceptor mRNA in arterial tissue was not changed by maternal L-NAME treatment. The mechanism of denervated arteries hypersensitivity downstream of the receptors may include membrane depolarization, and, consequently, the augmented activation of voltage-gated calcium channels [50] increased connexin 43 expression in smooth muscle cells and their electrical coupling [49], as well as the higher contribution of chloride channels to arterial contraction [14]. Further studies should show whether these mechanisms are also involved in increasing the adrenergic sensitivity of the sparsely innervated arteries of L-NAME offspring.

Thus, intrauterine L-NAME exposure reduces the density of sympathetic innervation of the saphenous artery. This may diminish the trophic influence of sympathetic nerves, and, consequently, cause a delay in arterial maturation, manifested in decreases in the inner diameter, maximal active force, and α-actin/β-actin expression ratio.

### 3.4. Intrauterine L-NAME Exposure Is Associated with the Weakened Sympathetic Control of Systemic Blood Pressure in Two-Week-Old Rats

Sympathetic nervous system activity contributes significantly to maintaining arterial pressure level. In rats, the sympathetic control of blood pressure levels has been observed from the ages of 1 to 2 weeks [7,8].

To identify whether the reduced density of sympathetic innervation observed in the saphenous artery of L-NAME offspring would be manifested at the systemic level, we performed the measurements of systemic hemodynamic parameters. We have shown, for the first time, that the mean arterial pressure levels in two-week-old L-NAME offspring are significantly lower in comparison to a control group. We have also demonstrated that differences in mean arterial pressure between the two experimental groups disappeared after ganglionic blockade by chlorisondamine.

The heart rate values in the two groups were similar both before and after the ganglionic blockade; therefore, the depressor effect of chlorisondamine primarily reflected the sympathetic influence on peripheral vascular tone. Therefore, the difference in arterial pressure levels between two groups of pups before, but not after, ganglionic blockade allows us to conclude that reduced blood pressure levels in L-NAME pups are due to a smaller neurogenic component of vascular tone.

Neurogenic components of blood pressure level are determined by the functions of sympathetic neurons (their discharge and neurovascular transmission) and vascular reactivity to the influence of neurotransmitters. Arterial responses to intermediate (“regulatory”) concentrations of the α_1_-adrenoceptor agonist were not reduced in two-week-old L-NAME pups; therefore, we suggest that smaller sympathetic components of vascular tone in L-NAME pups were due to the inhibition of sympathetic ganglionic neuron function because of their relative immaturity.

However, heart rate responses to the ganglionic blockade were not changed in L-NAME pups compared to control pups. Notably, the heart rate in rat pups is greatly influenced by adrenal catecholamines [53]. If adrenal chromaffin cells are less sensitive to developmental NO deficiency compared to ganglionic sympathetic neurons, this would explain unchanged heart rate responses to ganglionic blockade after intrauterine L-NAME exposure. Adrenaline has a higher potency to β-adrenoceptors compared to α-adrenoceptors [54]; therefore, its cardiostimulatory effect should be more prominent compared to the vasoconstrictor effect in 1–2-week-old pups.

Thus, intrauterine L-NAME exposure weakens the sympathetic control of peripheral vasculature in two-week-old rats. Importantly, our results suggest a weak development of sympathetic innervation in two-week-old L-NAME offspring to be typical not only for the saphenous artery, but also for other vascular regions.

## 4. Materials and Methods

### 4.1. Animals

Experiments were performed using Wistar rats in accordance with the European Convention on the protection of animals used for scientific purposes (EU Directive 2010/63/EU). All animal procedures were approved by the Moscow State University committee on animal welfare (protocol 133-g, approval date 2 July 2021). Sexually mature (3–4-month-old) male and female Wistar rats were obtained from the vivarium of the Research Institute of General Pathology and Pathophysiology, Russian Academy of Sciences (Moscow, Russia), and then bred in the laboratory animal unit of the Biological Faculty, Moscow State University. The animals were maintained with a 12/12 h light/dark cycle and had free access to normal rodent chow (Laboratorkorm, Moscow, Russia).

### 4.2. Rat Model of Intrauterine NO deficiency

Intrauterine NO deficiency was simulated by the suppression of NO production in the female body during the second half of pregnancy with the nitric oxide synthase inhibitor L-NAME (Chem-Impex International, Wood Dale, IL, USA). For mating, three females and two males were housed together overnight. The onset of pregnancy was determined by the presence of sperm in the vaginal smear the next morning (this day was considered GD1). On GD10, females were randomly assigned to control or L-NAME groups. Females in the L-NAME group received L-NAME in drinking water (at a concentration of 250 mg/L) from GD10 until delivery; females in the control group received tap water. During the pregnancy, the females of both groups were regularly weighed, and their water intake was recorded.

Female systolic BP and heart rate were also recorded using tail-cuff plethysmography (Systola, Neurobotics, Moscow, Russia). The first pre-pregnancy recording served to familiarize the animals with the procedure. During the pregnancy, the recordings were performed on GD7, GD15, and GD20. At every time point, the measurements were performed at least 5 times for each rat, and the obtained values were averaged. On GD18–GD19, blood samples (about 300 µL) were taken from the incision of females’ tail tips to determine biochemical parameters and the content of NO metabolites.

On the day of delivery (considered as PND1), the number of pups in each litter was limited to eight. Trunk blood was collected from one pup per litter (both sexes) for further analysis at PND2. The litters were regularly weighed from PND2 to PND14. Male pups aged 14 to 16 days old were further used in experiments on isolated arteries and blood pressure recordings.

### 4.3. Measurement of Blood Parameters

To obtain serum, blood samples were kept for 20 min at room temperature, and for an additional 40 min at +4 °C, and then centrifuged at 1350× *g* for 15 min. Serum was collected and stored at −20 °C until further analysis.

The concentrations of total cholesterol and triglycerides were measured by enzymatic colorimetric methods using reagents from Hospitex Diagnostics (Moscow, Russia). The determination of NO metabolite contents was carried out using the Griess method after the deproteinization of the samples, extraction of lipids, and the reduction of NO_3_^−^ to NO_2_^−^ using VCl_3_ simultaneously with the Griess reaction [55]. Optical density was measured spectrophotometrically at 540 nm (for total cholesterol and nitrites) or 510 nm (for triglycerides) using Multiskan EX (Thermo Electron Corporation, Langenselbold, Germany).

### 4.4. Visualization of Sympathetic Nerve Fibers

To visualize sympathetic nerves in the wall of the saphenous artery, we used a method based on the formation of a fluorescent complex resulting from the interaction of catecholamines with glyoxylic acid [30,56,57].

The saphenous arteries were isolated, carefully cleaned from the surrounding tissue, and then incubated in 0.1 M phosphate-buffered saline solution (pH 7.4) supplemented with glyoxylic acid (2%) and sucrose (10%) for 30 min at room temperature. Then, the arteries were flattened on a slide with the adventitia upward, dried (30 min in a jet of warm air and 5 min at 100 °C), and overlaid with mineral oil and a cover glass.

A confocal microscope Zeiss LSM700 with air Plan-Apochromat 20×/0.8 M27 objective (Carl Zeiss AG, Oberkochen, Germany) was used to visualize glyoxylic-acid-induced fluorescence in the immersed preparations. The emitted fluorescence was detected in confocal mode with a 0.56 μm pinhole in 405–480 nm (maximum at 435 nm) wavelength range and was induced by diode excitation with a 405 nm laser. Confocal 2048 × 2048 pixel images including 25 stacks that covered the entire thickness of tissue samples were recorded using Carl Zeiss ZEN 7.0 software.

Collected data were analyzed offline using ImageJ 1.50i software. Open-source Bio-Formats Explorer ImageJ plugins (imagej.net/Bio-Formats) were used to handle images. After background subtraction and binarization, skeletonization and skeleton analysis (imagej.net/Skeletonize3D) were performed in order to estimate the total length of catecholamine-positive fibers in the saphenous artery wall.

### 4.5. Functional Studies of Isolated Arteries

Saphenous arteries were carefully cleaned from the surrounding tissue, cut into 2 mm long segments, and mounted in a wire myograph (410A or 620M, DMT A/S, Aarhus, Denmark) for isometric force recording. The procedures of isolation and mounting were performed in a preparation solution containing: NaCl, 145 mM; KCl, 4.5 mM; CaCl_2_, 0.1 mM; MgSO_4_, 1.0 mM; NaH_2_PO_4_, 1.2 mM; EDTA, 0.025 mM and HEPES, 5.0 mM (pH = 7.4). The myograph chambers were heated to 37 °C and continuously bubbled with 5% CO_2_ in O_2_ to maintain pH at a constant level of 7.4. Data were recorded at 10 Hz using an analogue-to-digital converter (E14-140M, L-CARD, Moscow, Russia) and PowerGraph 3.3 software (DISoft, Moscow, Russia).

The standard start-up of each experiment included normalization procedures (Mulvany and Halpern, 1977) and activation. To achieve the full relaxation of arterial smooth muscle, normalization was performed in a calcium-free solution (containing: 120 mM NaCl, 26 mM NaHCO_3_, 4.5 mM KCl, 1 mM MgSO_4_, 1.2 mM NaH_2_PO_4_, 5.5 mM D-glucose, 0.1 mM EGTA and 5 mM HEPES) in the presence of 1 μM NO donor DEA/NO. Each arterial segment was stretched to 0.9d_100_ (90% of the inner diameter the segment would have at a transmural pressure of 100 mmHg), corresponding to the development of the maximum active force [58].

During activation and subsequent experimentation, arterial segments were kept in a solution containing: NaCl, 120 mM; NaHCO_3_, 26 mM; KCl, 4.5 mM; CaCl_2_, 1.6 mM; MgSO_4_, 1.0 mM; NaH_2_PO_4_, 1.2 mM; D-glucose, 5.5 mM; EDTA, 0.025 mM; and HEPES, 5.0 mM. The following substances were applied successively: (i) noradrenaline (10 μM, 3 min, twice); (ii) methoxamine (100 μM) for 5 min followed by acetylcholine (from 10 nM to 10 μM), to confirm the endothelium functionality; and (iii) methoxamine (10 μM). Noradrenaline, methoxamine and acetylcholine were obtained from Sigma (St. Louis, MO, USA).

Twenty minutes after the completion of the activation procedure, the concentration-dependent response to methoxamine was determined at the range from 0.01 to 100 μM of the drug. In some experiments, it was followed by a washing step and additional incubation with or without 50 μL of 100 μM L-NNA (N^ω^-Nitro-L-arginine, Alexis Biochemicals, San Diego, CA, USA). Subsequently, a second determination of the concentration-dependent response to methoxamine was performed at the same concentration range.

To calculate the active force values, the force value at the fully relaxed state was subtracted from all the measured values. Then, individual concentration–response relationships were fitted to a sigmoidal dose–response (variable slope) equation using GraphPad Prism 7.0 (La Jolla, CA, USA) to obtain pD2 (the negative logarithm of EC50) and E_max_ (maximum response). To estimate the effect of L-NNA on contractile responses, all active force values obtained during the second concentration-dependent response were expressed as the percentage of the maximum active force in the first concentration-dependent response.

### 4.6. Measurement of mRNA Content in Arterial Tissue by qPCR

Saphenous arteries were quickly isolated and placed in RNAlater^TM^ solution (Qiagen, Hilden, Germany) at −20 °C until the next process. Each sample included two arteries obtained from a two-week-old pup. RNA was extracted using the ExtractRNA Kit (Evrogen, Moscow, Russia), according to the manufacturer’s instructions, and processed with DNase I (Fermentas, 1000 U/mL). RNA concentrations were measured with a NanoDrop 1000 (Thermo Fisher Scientific, Waltham, MA, USA); then, all samples were adjusted to a concentration of 40 ng/μL. Reverse transcription was performed using the MMLV RT Kit (Evrogen, Moscow, Russia) according to the manufacturer’s instruction. qPCR was performed in the RotorGene6000 using qPCRmix-HS SYBR (Evrogen, Moscow, Russia). Primers used in this study were synthesized by Evrogen and are listed in Table 4.

mRNA expression levels were calculated as E^−Ct^, where E is the primer efficiency and Ct is the cycle number at which the product fluorescence rose above the threshold level. These values were normalized to the expression value for housekeeping gene *Rplp0*, detected in the same sample. All values were expressed as the percentage of the mean value in the control group.

**Table 4:** Gene-specific primers used in qPCR.

Protein	Gene	Forward	Reverse
α-Actin	*Acta2*	CCTGACCCTGAAGTATCCGA	CATCTCCAGAGTCCAGCACA
β-Actin	*Actb*	CAGGGTGTGATGGTGGGTATGG	AGTTGGTGACAATGCCGTGTTC
SM-MHC	*Myh11*	TTTGCCATTGAGGCCTTAG	GTTCACACGGCTGAGAATCCA
NM-MHC	*Myh10*	TGAGAAGCCGCCACACATC	CACCCGTGCAAAGAATCGA
α_1a_-adrenoceptor	*Adra1a*	GTAGCCAAGAGAGAAAGCCG	CAACCCACCACGATGCCCAG
RPLP0	*Rplp0*	AGGGTCCTGGCTTTGTCTGTGG	AGCTGCAGGAGCAGCAGTGG

### 4.7. Blood Pressure Measurement

Hemodynamic measurements were performed in urethane-anesthetized two-week-old pups (1.2 g/kg, i.p.). Successful anesthesia throughout the experiment was indicated as the absence of pupil and flexion reflexes with a potentially painful effect on the foot.

For arterial BP measurements, a polyethylene catheter (PE5-PE50) was inserted into the right carotid artery. To avoid clotting, the arterial catheter was continuously flushed with heparinized saline (50 U/mL) using a syringe pump (Model 341, SAGE Instruments, CA, USA); the infusion rate was 0.08 mL/h. For drug injections, a catheter (PE5-PE50) was inserted into the right jugular vein. Body temperature was maintained at 36–37 °C with a heating blanket throughout the experiment.

Arterial BP was continuously recorded at 1000 Hz using a BLPR2 transducer (World Precision Instruments, Sarasota, FL, USA) and a USB-6211 analog-to-digital converter (National Instruments, Austin, TX, USA). Data recording and processing were performed using custom-made software written in LabVIEW 2011 (National Instruments, Austin, TX, USA).

During the initial 15–20 min, arterial pressure and heart rate were allowed to stabilize. Then, the ganglionic blocker chlorisondamine (2.5 mg/kg, i.v.) was injected through a venous catheter, and a 15 min recording was obtained. The blood pressure signal was processed to estimate the mean arterial pressure and heart rate in each cardiac cycle. Hemodynamic parameters were averaged in time intervals taken before chlorisondamine administration (4 min, baseline) and 10 min after chlorisondamine administration (2 min).

### 4.8. Statistical Analysis

Statistics were calculated using GraphPad Prism 7.0 software (La Jolla, CA, USA). The normality of data distribution was confirmed using the Shapiro–Wilk test. Data are presented as the mean ± S.E.M. The differences between the two groups were assessed using the Student’s *t*-test or two-way ANOVA. Differences were considered statistically significant at *p* < 0.05; *n* is the number of rats in the group.

## 5. Conclusions

In conclusion, our novel findings demonstrate that NO deficiency in early ontogeny reduces the density of the adrenergic nerve plexus in peripheral arteries. This weakens sympathetic control of vascular tone and reduces the trophic influence of sympathetic nerves, which in turn causes structural and functional remodeling of the peripheral vasculature.

It should be mentioned that NO synthase inhibition is an established animal model of preeclampsia [25,26]. In our study, pregnant females supplemented with L-NAME have common symptoms of human preeclampsia, such as arterial hypertension, a decrease in the blood content of nitric oxide metabolites, and intrauterine growth restriction of the offspring [21,22,23]. Therefore, our findings could be useful for understanding the mechanisms of cardiovascular disorders in the offspring produced under conditions of maternal preeclampsia. In particular, the results of our study suggest impaired sympathetic control as a mechanism of a decreased arterial pressure level in human neonates born from women suffering from preeclampsia [24].

## Figures and Tables

**Table 1:** Serum parameters of control (CON) and L-NAME-treated females.

Parameters	CON	L-NAME
NOx, µM (*n* = 6; 6)	24.2 ± 2.0	16.3 ± 1.5 *
Triglycerides, mM (*n* = 6; 6)	3.32 ± 0.46	3.23 ± 0.25
Total cholesterol, mM (*n* = 6; 6)	1.95 ± 0.19	2.01 ± 0.13

Data are presented as the mean ± S.E.M., *n*, number of females. * *p* < 0.05 vs. control (unpaired Student’s *t*-test).

**Table 2:** Serum parameters of pups in control (CON) and L-NAME groups on the second and the 14th postnatal days.

Parameters	CON	L-NAME
Two-day-old offspring		
NOx, µM (*n* = 6; 6)	70.5 ± 5.4	17.3 ± 2.7 *
Triglycerides, mM (*n* = 6; 6)	1.62 ± 0.34	1.32 ± 0.35
Total cholesterol, mM (*n* = 6; 6)	2.21 ± 0.28	1.61 ± 0.20
14-day-old offspring		
NOx, µM (*n* = 6; 6)	22.3 ± 2.1	23.0 ± 2.7
Triglycerides, mM (*n* = 6; 6)	1.73 ± 0.18	2.46 ± 0.42
Total cholesterol, mM (*n* = 6; 6)	3.83 ± 0.23	3.70 ± 0.15

Data are presented as the mean ± S.E.M., *n*, number of animals. * *p* < 0.05 vs. respective control (unpaired Student’s *t*-test).

**Table 3:** Results of concentration–response relationship approximations of control (CON) and L-NAME offspring to a sigmoidal dose–response equation.

Parameters	CON	L-NAME
pD2 (*n* = 18;17)	4.82 ± 0.06	5.11 ± 0.05 *
Emax, mN (*n* = 18;17)	11.5 ± 0.4	9.1 ± 0.3 *

pD2, the negative logarithm of EC50 (the concentration of methoxamine which causes a half-maximum effect); E_max_, maximum response to methoxamine; *n*, number of animals. Data are presented as the mean ± S.E.M. * *p* < 0.05 vs. respective control (unpaired Student’s *t*-test).

## Data Availability

All the data generated during this study are available from the corresponding author on reasonable request.

## Supplementary Materials

The following are available online at https://www.mdpi.com/article/10.3390/ijms222212327/s1.
