# Supplementary material for: Intrauterine L-NAME Exposure Weakens the Development of Sympathetic Innervation and Induces the Remodeling of Arterial Vessels in Two-Week-Old Rats
Source: Int J Mol Sci. 2021 Nov 15;22(22):12327. doi: 10.3390/ijms222212327 (PMC8618620; doi:10.3390/ijms222212327)
Supplement: Supplementary file 1 [file ijms-22-12327-s001.zip › ijms-1432832-supplementary.pdf]

SUPPLEMENTARY MATERIALS

Intrauterine L-NAME Treatment Weakens the Development of Sympathetic Innervation and Induces Remodeling of Arterial Vessels in Two-Week Old Rats

Ekaterina K. Selivanova[1,†], Anastasia A. Shvetsova[1,†], Anna A. Borzykh[2], Dina K. Gaynullina[1], Oxana O. Kiryukhina[3], Elena V. Lukoshkova[4], Viktoria M. Potekhina[1], Vladislav S. Kuzmin[1], Olga S. Tarasova[1,2,*]

[1] Department of Human and Animal Physiology, Faculty of Biology, M.V. Lomonosov Moscow State University, 119234 Moscow, Russia; selivanova@mail.bio.msu.ru (E.K.S.); anastasiashvetsova92@gmail.com (A.A.S.); dina.gaynullina@gmail.com (D.K.G.); ku290381@mail.ru (V.S.K.); vm-potekhina@yandex.ru (V.M.P.)
[2] Laboratory of Exercise Physiology, State Research Center of the Russian Federation-Institute for Biomedical Problems, Russian Academy of Sciences, 123007 Moscow, Russia; borzykh.anna@gmail.com
[3] Laboratory for the Study of Information Processes at the Cellular and Molecular Levels, Institute for Information Transmission Problems, Russian Academy of Sciences, 119333 Moscow, Russia; kcyu@yandex.ru
[4] Laboratory of Experimental Pathology of the Heart, National Medical Research Center of Cardiology, Ministry of Health of the Russian Federation, 121552, Moscow, Russia

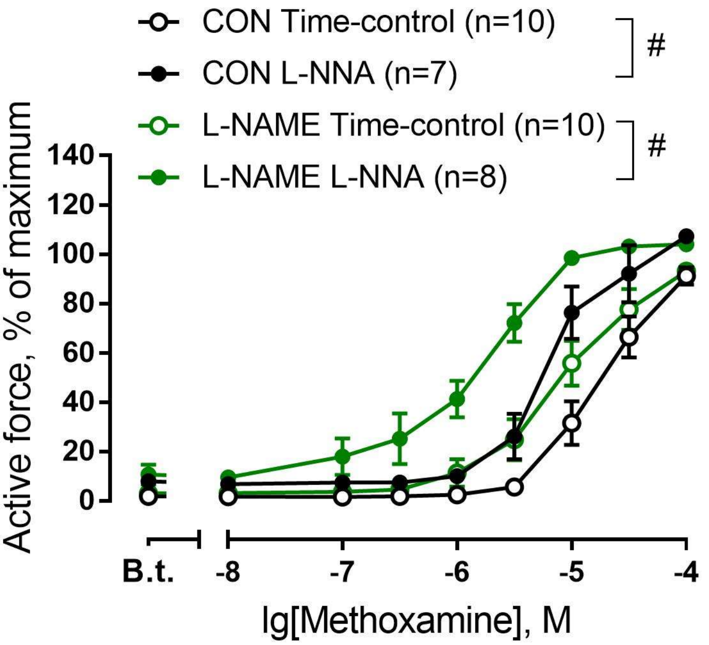

Figure S1. NO-synthase inhibitor did not remove the difference in saphenous artery contractile responses to methoxamine between pups from control (CON) and L-NAME offspring. Concentration-response relationships to α_1-adrenoceptor agonist methoxamine in the presence of solvent (H_2O, 50 µL per 5 mL myograph chamber, Time-control) or NO-synthase inhibitor L-NNA (100 µM) of saphenous arteries from control and L-NAME offspring, n – number of animals. B.t.—basal tone value (active force before the first concentration of methoxamine). Data are presented as mean and S.E.M. #$p<0.05$ (Repeated Measures ANOVA).
